# The Cotton Famine

**Published:** 1863-04

**Authors:** 


### Art. VI.—THE COTTON FAMINE.

Among the many miseries which have affected the human race—the painful disappointments which have rebuked their most sanguine hopes; the heavy losses which have distressed their family condition, and destroyed their domestic enjoyments; the bitter woes that have bowed down their bodily health, and the numerous trials which have too often prostrated their mental powers—may be ranked the sufferings of the operatives in the cotton towns of Lancashire and Cheshire.

Without entering into the political aspect of the American war, or stopping to minutely investigate the causes of the present lamentable condition of the cotton districts from the deficiency of the raw material, arising from the folly of depending for many years upon one source of supply of the staple for one of the largest branches of the national industry, which, long since, had been foreseen, and many warnings given, and therefore ought to have been prevented—but trade always attends to what is immediate, and cares little for consequences which are only probable—we will pass on to notice some of the effects and results of this dire calamity.

The elaborate Report of Dr. Buchanan, now before us, is full of "interest, not only to the profession, but to all classes and conditions of society."

The sad statements of the want of employment and loss of means of living to the operatives, and the tradesmen dependent upon their custom, the diseases induced by the want of comforts and insufficiency of nutriment, are of a most serious character, to which we might add the waste of capital among the employers.

From Dr. Buchanan's Report it appears that "a population of 2,000,000 has been for several months cut off from an adequate supply of the material upon which their prosperity depends. Nearly half a million of this class are cotton operatives, another half-million are directly dependent upon their labours, and the welfare of the remaining half of the population is to a great extent involved in the fortune of their neighbours. Taking the cotton districts as a whole, their state may be thus exhibited: Of every twenty-four persons, six are cotton workers, who habitually support other six. Of the six cotton workers, three are now earning nothing, two are earning part wages, *one* only is at full work. Of the six persons dependent upon them, one only could now be supported up to the ordinary mark. The remaining twelve persons, whose welfare is indirectly dependent on the cotton trade, will consist of about four persons who are earning, seven who are dependent on those four, and perhaps one who is a pauper. Of the four who are earning, only two have their usual incomes. One cotton worker, then, and two other persons, or three in all, are the only people out of the twenty-four who retain their full power of earning."

From the Report of Mr. Farnall, one of the Poor-law Inspectors, to the Central Executive Committee, it appears that the weekly returns of Poor-law relief in the twenty-seven distressed Unions up to the 6th of December last had shown a steady increase of dependence upon parochial aid. The whole number dependent on the poor-rate up to that date was 271,983 persons, which number happily was reduced on the 27th of that month to 260,506. The total cost of out-door relief at the latter date being 17,934*l*. 5*s*. 8*d*. against 3230*l*. 16*s*. 9*d*., compared with the correspondent week of the previous year—a heavy burden upon the ratepayers of those districts. The question here arises as to the wisdom of the cotton mills being confined to these districts; had they been dispensed throughout the country, the pressure upon the ratepayers would have been comparatively light. Under this heavy burden of accumulated woes, the intelligence of the operatives has been made more conspicuous in their manly, patient, *nay*, almost superhuman fortitude, whilst enduring inevitable evil, not from any act or cause of their own, but from causes over which they had no control, and which, under the existing circumstances, could not be averted.

Nor can we pass over the unparalleled sympathy their sufferings have awakened in every heart, from the highest in station to the humblest classes of society, and the fine spirit of philanthropy that has been developed, and the noble and almost unbounded spirit of benevolence which has been aroused and nourished throughout the community, and which in itself is an evidence of the advancement of true national greatness.

The appearance of typhus fever of the true Irish type, loss of strength, colour, and flesh, more especially among mothers who starve for their children's sake, intractable diarrhœa, dysentery, a hæmorrhagic tendency, purpura, actual scurvy, and a disposition in some cases to ulcerate and slough, consequent upon altered diet and extreme destitution, are some of the effects of the cotton famine mentioned by Dr. Buchanan. The Report of the Central Executive Committee, dated January 19th, 1863, states:—"Your Committee has now reached the middle of winter without the outbreak of any serious epidemic, or the appearance of any of those grave forms of disease, such as scurvy, diarrhœa, dysentery, which commonly follow prolonged low diet with little variety of foot, and the use of coarse and inferior kinds of aliment."

On referring to the Registrar-General's Report for the quarter ending December, 1862, we find that in the beginning of October typhus made its appearance in the sub-district of Preston, and proved fatal in 48 cases out of 227. In Manchester there were about 100 persons attacked with this fever, of whom 20 died.

"The existence of typhus fever," observes Dr. Buchanan, "suggests by itself doubts as to the complete success of the measures that have been adopted for the relief of the distressed. But on inquiry being made into the circumstances under which this disease prevailed in Preston and elsewhere, the existence of other morbid conditions was detected, which tended to corroborate such doubts. Some of these conditions consisted in a simple decline from the normal standard of health and strength, while others constituted positive disease."

While it is evident that typhus made its appearance in the towns mentioned, it must not be inferred from this that it has existed as an epidemic in the distressed cotton districts—indeed, it is gratifying to learn that in Chorlton-upon-Medlock that neither epidemic nor indeed any disease resulting from privation has prevailed among the unemployed operatives. From the Registrar-General's Quarterly Report for December, it is manifest that the high rate of mortality is not attributable to destitution arising from want. Scarlatina prevailed to a considerable extent in Manchester, Ashton-under-Lyne, and Oldham-above-Town, "affecting to a very considerable extent the mucous membranes; very bad throats, with nasal discharges and glands in the neck greatly swollen, have been commonly observed, and the mortality from this disease has been considerable, not only among the working class, but the general population. On reference to the Registrar-General's Reports, we find that scarlatina as an epidemic was conspicuous in the great seats of the cotton manufactories in 1848, and that it and other infectious diseases were rendered much more extensive by the utter want of ventilation in the dwellings of the poor.

The mortality in the North-western counties during the quarter ending September was peculiarly low.

During the succeeding quarter, bronchitis and pneumonia were, as might be expected, prevalent during the inclemency of November, aggravated from the want of proper clothing and bedding. Phthisis, although habitually in excess among this class of operatives, has not unusually manifested itself. Pertussis has been complicated with pneumonia.

Psora and diseases of the eczema and impetigo class have been rife from want of cleanliness; varicella in some towns, and rubeola in others, have been epidemic. The ordinary maladies of children have been very lightly felt, attributable to the greater care bestowed on them by their unemployed mothers instead of hired nurses. The fearful mortality among children of this class of operatives is too notorious to need any comment. In addition to maternal neglect, the administering to them of Godfrey's cordial, syrup of poppies, and other narcotics swells the death returns.

Drunkenness, with all its attendant evils and baneful consequences, is, with the exception of Manchester, Salford, and Wigan, less.

Venereal appears to be in excess.

As regards the existing state and habits, income and expenditure of the poor, Dr. Buchanan has gone very closely into the inquiry, and has fully reported the interesting results of his investigation. The pressure of the distress has been most felt in the towns *exclusively* dependent on the cotton trade. The change from a very generous diet to one of an opposite character has produced a very different standard of health among the unemployed cotton-workers Their average income from all sources ranges from 1*s.* 6*d.* to 2*s.* per head. Low health and actual disease has resulted, and will result, from their income being insufficient to procure them the animal and other food to which they have been accustomed. Bedding, in many instances, has been parted with; houses are becoming overcrowded, firing has been deficient, and the results have been, that numbers have huddled together as closely as possible for warmth, carefully excluding from their crowded sleeping apartments every breath of air.

This is a grave matter, and one to which it is hoped the local authorities have ere now directed their attention. House to house visitation ought to be vigorously and systematically carried out; stifling, pent-up, vitiated atmosphere made to give place to free ventilation and solar light; proportionate space assigned to each individual, drains attended to, and closets well supplied with water, together with all necessary sanitary measures; and last, though not least, a sufficient supply of good food and pure water, together with suitable bedding and clothing. The ventilation of sewing schools, and avoiding their being overcrowded, will save many of the young women employed in them from wasting and protracted sickness, and becoming untimely tenants of the grave.

Sad and lamentable as is and has been the cotton famine, may we not look upon it as likely to produce ultimately beneficial results? Space will only permit us to glance at a few of them.

The dependence of the cotton industry for the raw material will be extensively distributed. Egypt, Turkey, India, China, the West India islands, Queensland, and South Africa will furnish supplies. It will render us independent of the slaveholders. A better feeling will spring up among all classes in this country in consequence of the generous sympathy which the wealthy have exhibited for the working classes. Attention will be drawn to the best methods of alleviating the sufferings of the cotton artisans in their normal state. The working classes will have established a claim to political recognition. It will give much clearer views of the causes which affect the health of the people. Already has the beneficial effect of the fresh air and enforced temperance been felt by the unemployed workpeople, and diseases avoided, and children have been better nursed and cared for. In the darkness of the cotton famine a light has shined. The mildness of the winter has been a great advantage; a severe one, such as we had two years ago, would have aggravated the sufferings of the unemployed operatives, especially the sick, the aged, and the very young. Thus in judgment does the all-wise and beneficent Ruler of events remember mercy.

---

